# Predicting positive organizational behavior based on structural and psychological empowerment among nurses

**DOI:** 10.1186/s12962-021-00289-1

**Published:** 2021-07-02

**Authors:** Faranak Jafari, Nader Salari, Amin Hosseinian-Far, Alireza Abdi, Niaz Ezatizadeh

**Affiliations:** 1 Department of Nursing, School of Nursing and Midwifery, Kermanshah University of Medical Sciences, Kermanshah, Iran; 2 Department of Biostatistics, School of Health, Kermanshah University of Medical Sciences, Kermanshah, Iran; 3 Department of Business Systems and Operations, University of Northampton, Northampton, UK

**Keywords:** Positive organizational behavior, Empowerment, Structural empowerment, Psychological empowerment, Nurses

## Abstract

**Background:**

Positive Organizational Behavior (POB) as an application of positive psychology provides the opportunity to nurses to deliver an effective and high-quality service. This study aims to predict positive organizational behavior based on structural and psychological empowerment among Nurses.

**Method:**

In this descriptive-analytical study, the selected population was the nurses working in university hospitals affiliated to Kermanshah University of Medical Sciences, of which 152 people were selected using quota sampling strategy and according to the set entry criteria. The research data were collected using the standard questionnaires of Kanter’s Structural Empowerment, Spreitzer’s Psychological Empowerment, and Luthans’ Positive Organizational Behavior, and were then entered into SPSS16 software.

**Results:**

There are significant and direct relationships between the elements of structural empowerment with positive organizational behavior (r = 1.496), and psychological empowerment with positive organizational behavior (r = 1.379). Overall structural and psychological empowerment criteria predict 29% of variance in positive organizational behavior among nurses.

**Conclusion:**

This study highlights the importance of structural and psychological empowerment as strong predictors for positive organizational behavior.

## Background

Human capital in every organization is one of the most valuable resources that can allow firms to further develop their other key assets. To improve this capital, it is necessary to examine the internal dimensions of human beings that can influence organizational performance. One of the new topics of interest in human resource management subject area is positive organizational behavior. Positive organizational behavior was introduced by Fred Luthans. He believed that the study of the positive points of organizational behavior of human resources and their psychological capacities are effectively used in the management of performance improvement in organizations [1–3].

In the past, many psychologists and researchers in the field have mostly focused on the weaknesses of employees with respect to set organizational goals, and have proposed appropriate strategies to reduce such weaknesses [4].

Yet there has been less attention to the capabilities and positive aspects of employees’ behavior. With the emergence of the positive psychology subject area, the policy of making optimal use of positive capacities, attitudes, and human resource capabilities was introduced [4–6].

One of the indicators of positive psychology is psychological capital, which is defined as a person’s belief in his/her ability to achieve success, determination in pursuing goals, and creating positive collections about himself/herself, and enduring problems [7], Luthans has characterized psychological capital by the elements of self-efficacy, optimism, hope, and resilience [7].

In fact, the most important asset of organizations and the agent of achieving the goals and programs of any organization are the people who work in that organization. Experts believe that it is impossible to achieve organizational goals without skilled, committed and satisfied human resources [2–4].

In order for employees to have a positive attitude towards their job and organization, it is necessary to change their beliefs, thoughts and attitudes [8, 9].

If they believe that they have the ability and competence to perform tasks successfully, and also feel that they have the ability to influence and control job results, they will pursue valuable career goals whilst they believe that they will be treated transparently and fairly [10–12].

To empower employees, there are two categories of empowerment: structural and psychological. Structural empowerment involves adjusting workplace structures by managers and facilitating staff access to organizational facilities, as proposed by Counter (1993–1997). Moreover, structural empowerment is related to the delegation of power and authority in an organization [13].

Counter argues that structural empowerment is achieved through providing employees with the opportunity to have access to four environmental factors, namely, opportunity, information, support, and resources [13, 14]. Psychological empowerment, according to Spritzer’s definition, is a reflection of employees’ perceptions of control over their work environment. Spritzer’s dimensions of psychological empowerment are introduced as impact, meaning, competence, and self-determination [15, 16].

The results of several studies highlight the effects of positive organizational behavior on improving variables such as job desire [17], peace of mind [18], quality of working life [1, 19] and employee satisfaction and performance in the organization [9, 20].

Counter and other researchers in the area of structural empowerment also argue that increasing the understanding of structural empowerment in employees can increase organizational commitment, job satisfaction, and service quality among nurses. Additionally, psychological empowerment can lead to job satisfaction in nursing staff [13, 14].

However, no study has been conducted to predict dimensions of structural and psychological empowerments with positive organizational behavior [15, 16]. Considering the high occupational burnout and absenteeism from work among nurses due to the stressful nature of their work environment, it is beneficial to study the factors that predict their organizational behavior [17, 18].

Moreover, it is necessary to predict positive organizational behavior following structural and psychological empowerment as effective interventions [5]. Accordingly, this study was conducted with the aim of predicting positive organizational behavior based on the dimensions of structural and psychological empowerment among nurses.

## Methods

This is a descriptive-analytical (cross-sectional) study of positive organizational behavior among nurses working in university hospitals affiliated to Kermanshah University of Medical Sciences. A sample of 180 nurses from the above community was selected following a quota sampling strategy. Subsequently, 28 distorted and incomplete questionnaires were excluded, and responses from 152 nurses (59 men and 93 women) were used for our analysis.

### Determining the sample size

The sample size was determined using the correlation between the two quantitative variables in the study population, with 95% confidence (α-1) and 90% power of study (β1-), as well as taking into consideration the studies of Roshanzadeh et al., Bonyad Karizme et al., and Parastar et al. These studies have reported the relationship between psychological empowerment and psychological stress in nurses as 0.93 with respect to self-efficacy, 0.28 for job satisfaction, and 0.5 in relation to occupational burnout, respectively (6-7-9). Considering the above, the minimum required sample size was estimated as 120 participants. In view of the multiplicity of the questions, the criteria for including a participant’s response (questionnaires with 90% of the questions responded should be included), and the probability of response rate (50%), a total number of 180 participants were selected for this study.

The correlation of psychological empowerment with psychological stress based on Roshanzadeh’s study [17] is calculated as:

$$C = 0.5 \times Ln\left[ {\tfrac{{\left( {1 + \,r} \right)}}{{\left( {1 - r} \right)}}} \right],$$

$$n = \left[ {\frac{{Z_{\alpha } + Z_{\beta } }}{C}} \right]^{2} + 3,$$

$$r = 0.93,$$

$$\begin{aligned} C& = 0.5 \times Ln\left[ {\tfrac{{\left( {1 + r} \right)}}{{\left( {1 - r} \right)}}} \right] = 0.5 \times Ln\left[ {\tfrac{{\left( {1 + (0.93)} \right)}}{{\left( {1 - (0.93)} \right)}}} \right] \\ & = 0.5 \times Ln\left[ {19} \right] = 0.5 \times \left[ {2.94} \right] = 1.47 \simeq 1.5, \\ \end{aligned}$$

$$Z_{\alpha } = Z_{{0.95}} = 1.96,$$

$$Z_{\beta } = Z_{{0.90}} = 1.28,$$

$$\begin{aligned} n = & \left[ {\frac{{Z_{\alpha } + Z_{\beta } }}{C}} \right]^{2} + 3 = \left[ {\frac{{1.96 + 1.28}}{{1.5}}} \right]^{2} + 3 \\ & = 4.66 + 3 = 7.66, \\ \end{aligned}$$

$$n \ge 8.$$

Moreover, the correlation of psychological empowerment with structural empowerment based on the study conducted by Bonyad Karizme [13] is:

$$r = 0.28,$$

$$\begin{aligned} C& = 0.5 \times Ln\left[ {\tfrac{{\left( {1 + r} \right)}}{{\left( {1 - r} \right)}}} \right] = 0.5 \times Ln\left[ {\tfrac{{\left( {1 + (0.28)} \right)}}{{\left( {1 - (0.28)} \right)}}} \right] \\ & = 0.5 \times Ln\left[ {1.77} \right] = 0.5 \times \left[ {0.57} \right] \\ & = 0.285 \simeq 0.3, \\ \end{aligned}$$

$$Z_{\alpha } = Z_{{0.95}} = 1.96,$$

$$Z_{\beta } = Z_{{0.99}} = 1.28,$$

$$\begin{aligned} n = & \left[ {\frac{{Z_{\alpha } + Z_{\beta } }}{C}} \right]^{2} + 3 = \left[ {\frac{{1.96 + 1.28}}{{0.3}}} \right]^{2} + 3 \\ & = 116.64 + 3 = 119.64, \\ \end{aligned}$$

$$n \ge 120.$$

### Inclusion and exclusion criteria

Criteria for entering the study include the participant to hold a first, or a master’s degree in nursing, with clinical work experience for at least 2 years. Moreover, to be included, participants should have been willing to voluntarily partake in the research and should have been employed in one of the university hospitals in Kermanshah University of Medical Sciences.

#### Data collection methods

Three questionnaires were used to gather the required data, and these are discussed below.

### Psychological empowerment questionnaire

To measure the psychological empowerment perception of the nurses under study, the Spreitzer’s Psychological Empowerment Questionnaire (1984) was used. This questionnaire has 12 questions, and four dimensions that are impact, meaning, competence, and self-determination. Questions 1–3 are related to impact, 4–6 to meaning, 7–9 to competence, and questions 10–12 are related to self-determination. The participants can provide responses as per the five-part Likert scale (I completely disagree with a score of one, I disagree with a score of two, I have no opinion on a score of three, I agree with a score of four, and I completely agree with a score of five). To obtain the score for each dimension, the total score of the questions related to that dimension was collected. The minimum and the maximum overall scores across the four dimensions in this test are 12 and 60 respectively. A score of 12–24 indicates poor psychological empowerment perception, a score of 25–36 presents a moderate psychological empowerment perception, and a score of 37–60 demonstrates a strong psychological empowerment perception. In order to measure the reliability of the questionnaire, Cronbach’s alpha reliability coefficient (88%) was used; this reliability coefficient was also used in Bonyad Karizme et al. [13, 21].

### Structural empowerment questionnaire

To measure the structural empowerment among the participants, we used the 19-item Counter Conditions of Work Effectiveness Questionnaire (1977–1993)—CWEQ-2, which includes the dimensions of work effectiveness within items 1–12, opportunity to ask questions within items 1–3, information about questions in items 4–6, question support in items 7–9, the sources of questions in items 10–12, job activities scale (JAS) in items 13–15, and organizational relationship scale (ORS) in items 16–19. The answers could be selected from a five-point Likert scale (not at all as score one, slightly as score two, to some extent as score three, high as score four, and very high as score of five. To get the score for each dimension, we can add the average score of the questions related to that dimension. Similarly, to get the total score of the questionnaire, we can calculate the average score of each subgroup (there are six subgroups). The minimum overall score that can be obtained from the questionnaire is six and the maximum overall score is 30. Overall scores ranging from 6 to 13 show a low structural empowerment perception, 14–22 overall scores denote a moderate structural empowerment, and 23–30 represents a strong structural perception. The reliability of the questionnaire has been confirmed in the work of Fatemeh Parastar et al., with a Cronbach’s alpha of 79% [11, 14].

### Positive organizational behavior questionnaire

To evaluate positive organizational behavior, Luthans et al. [9] Psychological Capital Questionnaire (PCQ) was used. This questionnaire entails 20 items (questions) with four dimensions. The Self-efficacy dimension is assessed through questions 1–5, Hope with questions 6–10, Resilience using questions 11–15, and Optimism through questions 16–20. To get the score for each dimension, the sum of the scores of the questions related to that dimension is added together, and then to get the total score of the questionnaire, the sum of the scores of all the questions is summed up. A score of 20–40 shows a strong perception of positive organizational behavior, a score of 41–60 demonstrates a positive perception of moderate organizational behavior, and a score of 61–100 shows a strong perception of positive organizational behavior. The questionnaire is based on a five-choice Likert scale ranging from very low to very high, where one denotes very low, to five that represents very high. The minimum score that can be obtained from this test is 20 and the maximum is 100. A higher score indicates a better presentation of positive organizational behavior in the organization. The reliability and validity coefficients of the above-mentioned questionnaire (90% of Cronbach’s alpha reliability coefficient) have been confirmed in the research work of Davood Hosseinpour et al. [8].

### Statistical analysis

Then, in order to collect information, we first provided information to the research participants on how they could complete the questionnaires. They were also asked to provide honest answers to the questions. Then, they were provided with structural empowerment, psychological empowerment, and positive organizational behavior questionnaires. The collected data were entered into the SPSS16 statistical software. In this study, descriptive and inferential statistics were used to analyze the data. In the descriptive statistics section of scatter and central indices and in the inferential statistics section, in order to test the research hypotheses, Pearson correlation coefficient and multiple linear regression were used.

## Results

Considering to the statistical analysis of the gathered data, it was reported that 38.8% of nurses are male, 42.8% of participants had less than 30 years old, 93.4% had a bachelor’s degree, and 41.1% had less than 5 years of work experience (Table 1).

**Table 1:** Distribution of relative and absolute frequency of demographic variables in nurses

Variable	Frequency	Percentage
Gender		
Male	59	38.8
Female	93	61.2
Age (year)		
< 30	72	47.4
30–39	65	42.8
≥ 40	15	9.9
Education		
First degree	142	93.4
Master’s	10	6.6
Work experience		
Below 5 years	67	44.1
5 to 10 years	48	31.6
10 to 15 years	23	15.1
More than 15 years	14	9.2

Considering the results of the Pearson’s correlation coefficient test, presented in Table 2, there is a direct and significant relationship between the scale of positive organizational behavior (total score) and structural empowerment (r = 0.496 and P < 0.001).

**Table 2:** Pearson’s correlation test results between structural empowerment scale with positive organizational behavior and its subscales

Criteria	Self-efficacy	Hope	Resilience	Optimism	Positive organizational behavior
	r	r	r	r	r
	P-value	P-value	P-value	P-value	P-value
Opportunity for advancement	0.215	0.291	0.284	0.359	0.361
	0.008	0.001	0.001	0.001	0.001
Access to information	0.25	0.273	0.181	0.359	0.341
	0.002	0.001	0.026	0.001	0.001
Access to support	0.2	0.297	0.185	0.486	0.362
	0.014	0.001	0.022	0.001	0.001
Access to resources	0.157	0.335	0.218	0.35	0.336
	0.055	0.001	0.007	0.001	0.001
Formal power	0.204	0.277	0.113	0.378	0.301
	0.012	0.001	0.164	0.001	0.001
Information power	0.337	0.408	0.23	0.369	0.349
	0.001	0.001	0.004	0.001	0.001
Structural empowerment	0.321	0.437	0.278	0.528	0.496
	0.001	0.001	0.001	0.001	0.001

As shown in Table 3, the results of the Pearson’s correlation coefficient test demonstrated that psychological empowerment has a direct, positive and significant correlation with positive organizational behavior (r = 0.379 and P < 0.001).

**Table 3:** The results of Pearson’s coefficient correlation test between the psychological empowerment scale with positive organizational behavior and its subscales

Criteria	Self-efficacy	Hope	Resilience	Optimism	Positive organizational behavior
	r	r	r	r	r
	P-value	P-value	P-value	P-value	P-value
Impact	0.077	0.301	0.144	0.169	0.222
	0.384	0.001	0.076	0.037	0.006
Meaning	0.057	0.309	0.164	0.292	0.242
	0.483	0.001	0.043	0.001	0.003
Competence	0.178	0.384	0.362	0.311	0.369
	0.029	0.001	0.001	0.001	0.001
Self-determination	0.125	0.152	0.323	0.005	0.203
	0.126	0.055	0.001	0.952	0.012
Psychological empowerment	0.155	0.413	0.355	0.297	0.379
	0.057	0.001	0.001	0.001	0.001

According to the findings, the Adjusted R^2^ (adJR^2^) level in this model is 29%, and this is a prediction of the percentage of the positive organizational behavior variance in the respondents. In other words, the structural and psychological empowerment scales predict 29% of the positive organizational behavior variance in nurses.

The results presented Table 4 demonstrate the analysis of regression variance, according to which the F-value of the regression model is 31.6, whilst the P-value is small and is 0.001. Therefore, the regression model with two variables is significant.

**Table 4:** Results of multivariate regression variance analysis simultaneously in predicting positive organizational behavior in nurses

Model	The sum of the squares	Degree of freedom	Mean squares	F-value	P-value
Regression	6019.74	2	3009.8	31.6	0.001
Residual	14097.06	148	95.25		
Total	20116.8	150	–		

The results of multivariate regression analysis (using the enter method to enter variables independently) are presented in Table 5. The results show that the independent variables of structural empowerment and psychological empowerment have the most significant predictive power for the dependent variable of positive organizational behavior. This analysis denotes that structural and psychological empowerments predict 29% of the variance of the dependent variable of positive organizational behavior (P < 0.001, df = 148, F = 31.6). Moreover, by increasing the standard deviation in the structural empowerment score, the score of the positive organizational behavior of nurse’s increases by 0.496 of the standard deviation.

**Table 5:** The relationship between psychological and structural empowerments with positive organizational behavior based on the results of the multivariate regression test using the enter method

Predictor Variable	Unstandardized coefficients		Standardized coefficients	T	P-value	Confidence interval 95%
	SB	β				
Intercept	22.83	5.64	–	0.04	0.001	11.68–33.99
Psychological empowerment	0.418	0.125	0.244	0.35	0.001	0.172–0.655
Structural empowerment	0.157	0.080	0.417	0.73	0.001	0.299–0.614

R = 0.547

R^2^ = 0.299

ADJR^2^ = 0.29

Additionally, with the increase of a standard deviation in the psychological empowerment value, the score of positive organizational behavior in nurses increase by 0.244 of the standard deviation. Tables 3, 4, 5 illustrate such inference (please also see Table 5). As can be seen in Table 5, the model parameters include the intercept and the regression coefficients, and these coefficients for the variables of psychological empowerment and structural empowerment are 0.244 and 0.417 respectively. Therefore, the regression model can be defined as:

$${\text{Y}} = 22.8 + 0.244 \times 1 + 0.147 \times 2.$$

This model shows the positive relationship between psychological empowerment and structural empowerment variables with positive organizational behavior.

## Discussion

The general understanding of the structural empowerment of nurses in Kermanshah University of Medical Sciences university hospitals was average and in general all subdimensions (i.e., Opportunity for advancement, Access to information, Access to support, Access to resources, Formal power, and Information power) were also average. Studies of Charisma et al. [13], Eskandari et al. [22], Marie Ja et al. [23], Sandra et al. [24] and Maria et al. [25] have all interpreted the structural empowerment of nurses as average, which is also in line with our findings.

The existing literature argue that nurses’ perception of structural empowerment and its dimensions (especially access to opportunity, information, support and resources) in different countries and in different years is at an average level. Despite the importance of empowering employees and the impacts of this on improving the quality of services and reducing the costs of health systems, such empowerment has not yet been realized at the highest levels of health organizations.

In a study, Marie Ja et al. evaluated the level of psychological empowerment perception among nursing managers in Lithuania and reported it as high [26–28]. In another study in Tehran, Ghaniyoun et al. argued that the psychological empowerment of medical emergency personnel is moderate [29, 30]. Moreover, Hatamian et al. studied the psychological empowerment of middle-aged and elderly employees working in various organizations in Kermanshah and reported the empowerment mean as average [15, 31–49]. The reason for such a difference in the results of these pieces of research is probably related to heterogeneity of the selected research communities in each work.

In this work, the level of understanding of the positive organizational behavior of nurses in university hospitals of Kermanshah University of Medical Sciences was assessed as high. Moreover, we have found that there is a relationship between structural and psychological empowerment perceptions with positive organizational behavior in nurses of Kermanshah University of Medical Sciences university hospitals. Considering the findings, a positive and significant relationship was observed between structural scale of positive organizational behavior (total score) and structural empowerment. Psychological empowerment scale was found to be directly, positively and significantly correlated with positive organizational behavior. Besides, structural and psychological empowerment predicts 29% of the variance of the dependent variable of positive organizational behavior.

The findings show that there is a relationship between structural and psychological empowerment and positive organizational behavior in nurses. In this regard, Bonyad Karizmeh et al. examined the relationship between structural and psychological empowerment and job satisfaction of nurses in state hospitals in Mashhad, and concluded that there is a significant relationship between structural and psychological empowerment components with job satisfaction. Moreover, they demonstrated that three variables of Meaning, Access to Support, and Impact together predict 28.6% of job satisfaction variance [13, 50].

Nicoles et al. studied the effect of nurses’ structural empowerment on the quality of outcomes in hospitals. They concluded that structural empowerment is one of the key management practices and plays a key role within the nurses’ professional environments and the quality of outcomes in hospitals [3, 51–57].

Fang et al. examined the relationship between structural empowerment, psychological empowerment and emotional fatigue of nurses (through a meta-analysis) and concluded that there is an inverse relationship between structural and psychological empowerment with emotional fatigue in nurses [58], Jiajia et al. argued that there is an inverse relationship between job stress and burnout in nurses with structural empowerment. This study was conducted in China [12].

Hagerman et al. as part of a study which was conducted in Sweden, concluded that managers who have a strong access to structural empowerment were more likely to provide access to structural empowerment to their subordinate employees [59], while Regan et al. (2015) reported that empowerment, credible leadership, and professional practice have direct impacts on nurses’ understanding of interprofessional cooperation [24]. In two separate studies, it was reported that there is a positive relationship between structural empowerment and organizational commitment of nurses [60, 22].

Hartmann et al. in their research work in Australia, concluded that psychological empowerment increases behaviors related to climate protection that in turn result from understanding a personal responsibility. They went on to argue that psychological empowerment is a motivational structure in understanding preventive behavior [61, 30].

In a more recent study, Abdulrab et al. reported that psychological empowerment enhances the level of organizational citizenship behavior among employees, and is effective in increasing the quality of employees training [62]. Moreover, Hatamian et al. argued that there is a direct relationship between that one’s job satisfaction and psychological empowerment [15, 63].

The results observed in the literature are in line with the findings of our work. In literature, it has been argued that there is a direct relationship between structural and psychological empowerment with other variables such as: job satisfaction, quality of outcomes in hospital, nurses' understanding of interprofessional cooperation, organizational citizenship behavior, organizational citizenship behavior, nurses’ performance, organizational commitment of nurses, and job interaction. There is also an inverse relationship between structural and psychological empowerment with variables such as emotional exhaustion, job stress and burnout. Therefore, these findings strengthen the arguments in our work with respect to the relationship between positive organizational behavior, and structural and psychological empowerments.

The results from the above-mentioned studies show the importance of paying attention to the psychological capital of organizations, which may ultimately result in organizations gaining a competitive advantage. Moreover, considering today’s intense competitions in many sectors, there should not only a focus on the economic, human and social capital of an organization, but also, it is necessary to strongly consider psychological capital as a factor contributing to the survival of an organization in competitive markets. This type of capital is a valuable asset for organizations. Moreover, psychological capital positively impacts outcome and overall performance of an organization.

Given the theoretical underpinnings of our study and the importance of positive organizational behavior, increasing the positive organizational behavior of nurses and allowing nurses to grow and improve in hospitals can positively influence their performance.

## Limitations

Participants in this study were self-reporting as part of the questionnaire data collection method; therefore, the downsides of self-reporting data collection are integral part of the instrument, and this can be considered as one of the limitations of this work. Lack of access to all nurses working in the all provinces hospitals. Because the study is limited to nurses working in teaching hospitals affiliated to Kermanshah University of Medical Sciences, its generalizability must be done carefully.

## Conclusion

The structural and psychological empowerment of employees, can predict their positive organizational behavior by improving employees’ perceptions of structural and psychological empowerments, their perception of positive organizational behavior improves**.**

## Data Availability

Datasets are available through the corresponding author upon reasonable request.

## Abbreviation

POB: Positive organizational behavior
